# A minimal physiologically based pharmacokinetic model for high-dose methotrexate

**DOI:** 10.1007/s00280-021-04305-2

**Published:** 2021-06-13

**Authors:** Giuseppe Pesenti, Marco Foppoli, Davide Manca

**Affiliations:** 1 PSE-Lab, Process Systems Engineering Laboratory, Dipartimento di Chimica, Materiali e Ingegneria Chimica “Giulio Natta”, Politecnico di Milano, Piazza Leonardo da Vinci 32, 20133 Milano, Italy; 2 Unit of Lymphoid Malignancies, Division of Onco-Hematological Medicine, Department of Onco-Hematology, IRCCS San Raffaele Scientific Institute, Via Olgettina 60, 20132 Milano, Italy

**Keywords:** Methotrexate, HDMTX, Pharmacokinetics, Minimal PBPK, Renal function

## Abstract

**Purpose:**

High-dose methotrexate (HDMTX) is administered for the treatment of a variety of malignant tumors. Wide intra- and inter-individual variabilities characterize the pharmacokinetics of MTX, which is mostly excreted renally. HDMTX dosages are prescribed as a function of body surface area whereas dose adjustments depending on renal function are not well defined. We develop a population pharmacokinetic model with a physiological description of renal excretion as the basis for clinical tools able to suggest model-informed dosages and support therapeutic monitoring.

**Methods:**

This article presents a minimal physiologically based pharmacokinetic (PBPK) model for HDMTX, which specifically accounts for individual characteristics such as body weight, height, gender, age, hematocrit, and serum creatinine to provide individualized predictions. The model supplies a detailed and mechanistic description of capillary and cellular exchanges between plasma, interstitial fluid, and intracellular fluid compartments, and focuses on an individualized description of renal excretion.

**Results:**

The minimal PBPK model is identified and validated with a literature dataset based on Chinese patients suffering from primary central nervous system lymphoma. A comparison with a pharmacokinetic model from the literature suggests that the proposed model provides improved predictions. Remarkably, the model does not present any significant bias in a wide range of degrees of renal function.

**Conclusion:**

Results show that model predictions can capture the wide intra- and inter-individual variability of HDMTX, and highlight the role played by the individual degree of renal function. The proposed model can be the basis for the development of clinical decision-support systems for individualized dosages and therapeutic monitoring.

## Introduction

Methotrexate (MTX) is an antifolate drug used to treat auto-immune disorders and in cancer chemotherapy. High-dose methotrexate (HDMTX) consists of doses higher than 500–1000 mg/m^2^ [1, 2]. HDMTX treats a variety of malignant tumors, which include primary central nervous system lymphoma (PCNSL), osteosarcoma, and acute lymphoblastic leukemia [3]. HDMTX is used to extend the exposure duration, overcome the mechanisms of resistance to MTX [4, 5], and penetrate the blood–brain barrier [6].

The administration of HDMTX is characterized by a very large intra- and inter-individual variability [7–9] and is associated with toxic side effects, with a reported incidence of HDMTX-induced nephrotoxicity that goes from 1.8% [10] to 10.7% [11]. While there are several treatment protocols for each tumor type, they usually prescribe predetermined HDMTX dosages per square meter of body surface area (BSA) [2, 12–14].

MTX is mostly excreted through the kidneys [15] with creatinine clearance (CrCl) being a strong determinant of methotrexate overall clearance [5, 16]. Renal dysfunction leads to delayed MTX elimination and elevated plasma levels [10]. Despite this, adjustments for renal function are usually limited to the definition of cut-off values of either CrCl or glomerular filtration rate (GFR) for dose reduction or omission [1, 2, 4, 17]. Abrey [12] and Joerger and coauthors [16] report that uniform HDMTX dosing regimens are still lacking in the case of PCNSL, and suggest the introduction of personalized dosages to account for patient age, gender, and renal clearance, as they could significantly improve HDMTX treatment.

Few approaches to HDMTX dose adjustment have been published in the literature. Pignon et al. [18] proposed a dose adjustment approach to be performed during each 8-h cycle. Similarly, Evans et al. [19] showed that, in children with acute lymphoblastic leukemia, dose adjustments after 8 h of a 24 h MTX infusion lead to improved outcomes. Joerger et al. [9] proposed a dosing algorithm that uses the value of MTX concentration 24 h after the start of the first treatment cycle to adjust individual MTX doses in the following treatment cycles.

To the best of our knowledge, a decision-support tool capable of suggesting individualized HDMTX dosages for clinical use was not published in the literature. Accordingly, this article focuses on physiologically based pharmacokinetic (PBPK) modeling as the basis to develop that decision-support tool. Such a tool would also be suitable to support HDMTX therapeutic monitoring [20].

In 1970, Bischoff et al. [21] developed a remarkable pharmacokinetic model of methotrexate for mice, whose parameters attempted to maintain a good consistency with physiology. Then Zaharko et al. [22], Bischoff et al. [23], and Dedrick et al. [24] published developments of the same PBPK model for mice, other mammals, and humans. More recently, Ogungbenro et al. [25] proposed a methotrexate PBPK model for children and adults. However, these models do not account for individual characteristics such as body weight, height, gender, age, and degree of renal function, and their predictions of MTX pharmacokinetics can be directly applied only to a reference patient with standard body features.

We apply the general modeling approach proposed by Cao and Jusko [26] and present a new minimal PBPK model for the administration of intravenous HDMTX to adult patients that can account for individual characteristics such as body weight, height, gender, age, hematocrit, and renal function.

Model parameters are assigned as much as possible a priori and are functions of individual characteristics. The remaining parameters (which cannot be assigned a priori) are evaluated numerically via a non-linear regression against a literature dataset of patients suffering from PCNSL and via a bootstrap analysis. Our model adopts a population-PK approach, and the regressed parameters are assumed to constant for the whole population described.

The model devotes special attention to renal filtration. Consequently, it can be applied also to patients with reduced renal function. This approach allows improving the understanding of MTX pharmacokinetics, identifying the characteristics of peculiar populations, and refining the predictions of individual MTX pharmacokinetics.

## Methods

### Mathematical model

**Fig. 1:**
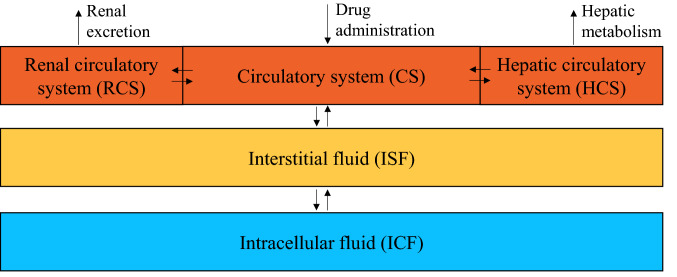
Structure of the minimal PBPK model. The rectangles represent the compartments of the model: three plasma compartments (dark orange), the lumped ISF compartment (mustard), and the lumped ICF compartment (cyan). The arrows describe the drug flows, *i.e.* administration, exchange between compartments, metabolism, and excretion (colour figure online)

Figure 1 shows the overall structure of our minimal PBPK model, which was implemented in MATLAB R2020b (The MathWorks, Natick, MA, US). The model adopts the general minimal PBPK approach proposed by Cao and Jusko [26] and describes two lumped compartments for interstitial fluid (ISF) and intracellular fluid (ICF). The plasma circulation of the drug is described by dividing plasma into the renal circulatory system (RCS), the hepatic circulatory system (HCS), and the remaining part of the global circulatory system (CS). Each compartment is assumed homogeneous [27] so that in the generic compartment $j$ with volume $V_{j}$, the drug concentration $c_{j}$ can be calculated as $c_{j} = {{m_{j} } \mathord{\left/ {\vphantom {{m_{j} } {V_{j} }}} \right. \kern-\nulldelimiterspace} {V_{j} }}$, where $m_{j}$ is the amount of drug.

The following system of ordinary differential equations (Eqs. 1–5) mathematically describes the drug material balances in the five model compartments (i.e. CS, RCS, HCS, ISF, and ICF) of Fig. 1:

1 $$\frac{{dm_{{CS}} }}{{dt}}\; = \;\dot{m}_{{IV}} - Q_{{CS \leftrightarrow RCS}} c_{{CS}} + Q_{{CS \leftrightarrow RCS}} c_{{RCS}} - Q_{{CS \leftrightarrow HCS}} c_{{CS}} + Q_{{CS \leftrightarrow HCS}} c_{{HCS}} - \dot{m}_{{CS \to ISF}} + \dot{m}_{{ISF \to CS}}$$

2 $$\frac{{dm_{RCS} }}{dt} = Q_{CS \leftrightarrow RCS} c_{CS} - Q_{CS \leftrightarrow RCS} c_{RCS} - \dot{m}_{excretion}^{renal} ,$$

3 $$\frac{{dm_{HCS} }}{dt} = Q_{CS \leftrightarrow HCS} c_{CS} - Q_{CS \leftrightarrow HCS} c_{HCS} - \dot{m}_{elimination}^{hepatic} ,$$

4 $$\frac{{dm_{ISF} }}{dt} = \dot{m}_{CS \to ISF} - \dot{m}_{ISF \to CS} - \dot{m}_{ISF \to ICF} + \dot{m}_{ICF \to ISF} ,$$

5 $$\frac{{dm_{ICF} }}{dt} = \dot{m}_{ISF \to ICF} - \dot{m}_{ICF \to ISF} ,$$

where $\dot{m}_{IV}$ is the MTX flow administered intravenously at each instant, $Q_{CS \leftrightarrow RCS}$ and $Q_{CS \leftrightarrow HCS}$ are the plasma flow rates exchanged with RCS and HCS, $\dot{m}_{CS \to ISF}$ and $\dot{m}_{ISF \to CS}$ represent the MTX flow rates exchanged between CS and ISF at the capillary level, $\dot{m}_{ISF \to ICF}$ and $\dot{m}_{ICF \to ISF}$ are the MTX flow rates entering and leaving ICF due to cellular exchange with ISF, $\dot{m}_{excretion}^{renal}$ is the renal excretion, and $\dot{m}_{elimination}^{hepatic}$ is the hepatic metabolic elimination. The model describes the capillary exchange between ISF and the plasma compartments as if it took place only within the global CS compartment.

### Plasma, ISF, and ICF volumes

Assuming that the body volumes grow linearly with body weight, the reference values of 5.3 L blood volume for a 73 kg man and 3.9 L for a 60 kg woman [28] allow inferring that men and women have 72 mL and 65 mL of blood per kg, respectively. Total plasma volume can be easily related to total blood volume by subtracting the hematocrit (HCT). The RCS and HCS plasma volumes are estimated, respectively, as 2% and 10% of total plasma [28]. Consequently, the CS plasma volume is the remaining fraction of plasma volume. The extracellular fluid (ECF) and ICF volumes are likewise estimated using as reference the average ECW and ICW values from Ritz et al. [29]. Finally, the ISF volume is calculated as the difference between ECF and plasma volumes.

Notably, our model does not fit the bodily volumes, and their values are instead assigned as individualized estimates from the patient’s body weight and HCT. All the volume estimates refer to the physiological volumes of plasma, ISF, or ICF. It should be noted, however, that these values do not take into account the individual excess fat, nutrition, race, and age, and may further change according to, e.g. hydration and sodium balance.

### Plasma flow rates

The total cardiac output (CO) $Q_{CO}^{plasma}$ (in mL/min) and the fractions of CO that reach the kidneys and the liver are estimated with the equations developed by Stader et al. [30] as a function of body weight, height, gender, age, and HCT.

### Methotrexate protein binding

MTX is affected by protein binding, *i.e.* the reversible binding between the drug and blood proteins [31]. Protein binding of MTX occurs predominantly with serum albumin [32] and the drug is inactive while bound.

In plasma, reported values of MTX protein binding for healthy patients usually fall in the 42–57% interval [31] and appear to decrease in patients that are ill or in remission (*e.g.* to 34–40% [33]). To apply the model to patients who suffer from tumor diseases and require the administration of HDMTX, the MTX bound fraction in plasma $f_{plasma}^{b}$ is assumed constant and equal to 42% [31]. Albumin gives rise to protein binding also in the ISF [34]. Following the approach of Schmitt [35], the MTX bound fraction in ISF $f_{ISF}^{b}$ is estimated as 28%. Therefore, the complementary MTX unbound fractions $f_{plasma}^{u}$ and $f_{ISF}^{u}$ are 58% and 72%, respectively.

### Methotrexate renal excretion

The main elimination route for MTX is renal excretion [2, 15]. Following intravenous administration of HDMTX, about 90% of the drug leaves the body unchanged through the urine within 154 h (*i.e.* almost a week) [36]. MTX is filtered by the renal corpuscles of each nephron, and undergoes active secretion and active reabsorption [15] as follows:

6 $$\dot{m}_{excretion}^{renal} = \dot{m}_{filtration} + \dot{m}_{secretion} - \dot{m}_{reabsorption} .$$

Assuming that unbound MTX is freely filtered across the glomerular filtration barrier and neglecting the filtration of albumin-bound MTX, renal filtration is described as

7 $$\dot{m}_{filtration} = Q_{GFR} c_{RCS} f_{plasma}^{u} .$$

Several equations have been developed in the literature to estimate the individual $Q_{GFR}$ from the serum creatinine levels, since obtaining experimental creatinine values from the blood is cheap and convenient. These equations correlate the blood creatinine levels to the steady-state values of $Q_{GFR}$ for a specific population, also by taking into account the body weight, height, gender, and age of the patient. In this work, we employed the CKD-EPI equation with a 4-level variable for race [37], which is suitable to describe both patients with renal impairment and with normal renal function. We used the coefficients for Asians to describe Chinese patients. Additionally, the 97.5th percentile of the reference interval by the FAS equation [38] was used as the upper limit to the $Q_{GFR}$ estimates.

Secretion and reabsorption are described as a single overall saturable process (Eqs. 8 and 9) with Michaelis–Menten kinetics. The modeling of reabsorption is simplified and is expressed as a function of the drug concentration inside the plasma, similar to secretion.

8 $$\dot{m}_{secretion} = \dot{m}_{secretion}^{max} \cdot \frac{{c_{RCS}^{u} }}{{K_{50}^{secretion} + c_{RCS}^{u} }},$$

9 $$\dot{m}_{reabsorption} = \dot{m}_{reabsorption}^{max} \cdot \frac{{c_{RCS}^{u} }}{{K_{50}^{reabsorption} + c_{RCS}^{u} }}.$$

The Michaelis constants $K_{50}^{secretion}$ and $K_{50}^{reabsorption}$ are estimated as 0.1 µmol/L and 0.6 µmol/L, multiplied by $f_{u,plasma}$ [15], whereas $\dot{m}_{secretion}^{max}$ and $\dot{m}_{reabsorption}^{max}$ are assigned as 5.189 and 1.038 µg/min (for a reference $Q_{GFR}$ of 100 mL/min, assuming a proportionality to $Q_{GFR}$). The overall MTX renal clearance described exceeds that of creatinine by up to 15% in the 0.1–0.4 µmol/L range and is consistent with the experimental trends [15]. As MTX concentration increases, secretion and reabsorption reach saturation and their relative contribution becomes smaller, so that renal clearance approaches creatinine clearance and the glomerular filtration rate.

### Methotrexate hepatic elimination

MTX is affected by uptake into the hepatocytes of the liver, where it is metabolized into its major metabolite, 7-hydroxymethotrexate (7-OH-MTX), and is also affected by biliary secretion. In the intestinal tract, MTX can be reabsorbed into the systemic circulation, excreted through the feces, or hydrolyzed to 2,4-diamino-N10-methylpteroic acid (DAMPA), a minor metabolite [39]. These routes represent a minor contribution to the overall MTX elimination [40], which does not appear correlated to biomarkers of liver function [41]. In the case of HDMTX, since these routes depend mostly on saturable processes, it is likely that their relative importance decreases further. Hepatic processes are therefore described with a simplified approach, which neglects the enterohepatic circulation and assumes that MTX affected by hepatic clearance is immediately either metabolized into 7-OH-MTX or secreted into the bile and metabolized into DAMPA or eventually excreted through the feces (Eq. 10).

10 $$\dot{m}_{elimination}^{hepatic} = Q_{clearance}^{hepatic} c_{HCS}^{u} .$$

The hepatic clearance is estimated as 4% of the GFR so that the overall hepatic elimination represents about 4% of the overall MTX clearance, approximately accounting for a 3% contribution from 7-OH-MTX metabolism [40] and 1% from DAMPA metabolism and excretion through feces [15].

### Methotrexate capillary exchange

MTX reaches the interstitial fluid through extravasation from the blood vessels at the capillary level. We describe capillary exchange in all tissues as generally permeability-limited (or diffusion-limited) [26, 42]. We assume that MTX is exchanged only by passive transport mechanisms, *i.e.* simple diffusion, according to the unbound $c_{CS}^{u}$ and $c_{ISF}^{u}$ concentrations:

11 $$\begin{gathered} \dot{m}_{CS \to ISF} = K_{CS \to ISF} c_{CS}^{u} , \hfill \\ \dot{m}_{ISF \to CS} = K_{ISF \to CS} c_{ISF}^{u} . \hfill \\ \end{gathered}$$

The exchange constants $K_{CS \to ISF}$ and $K_{ISF \to CS}$ represent the permeability-surface area product $PS$ (mL/min) and are estimated with Eq. (12) from their corresponding intensive constants $k_{CS \to ISF}$ and $k_{ISF \to CS}$ (min^−1^).

12 $$\begin{gathered} K_{CS \to ISF} = k_{CS \to ISF} V_{ISF} , \hfill \\ K_{ISF \to CS} = k_{ISF \to CS} V_{ISF} . \hfill \\ \end{gathered}$$

Concerning the exchange between CS and ISF, at steady-state $\dot{m}_{CS \to ISF} = \dot{m}_{ISF \to CS}$ and, therefore,

13 $$\frac{{c_{ISF}^{u} }}{{c_{CS}^{u} }} = \frac{{K_{CS \to ISF} }}{{K_{ISF \to CS} }} = \frac{{k_{CS \to ISF} }}{{k_{ISF \to CS} }},$$

14 $$\frac{{c_{ISF} }}{{c_{plasma} }} = \frac{{f_{plasma}^{u} }}{{f_{ISF}^{u} }}\frac{{k_{CS \to ISF} }}{{k_{ISF \to CS} }}.$$

By defining the tissue partition coefficient as ${{c_{ISF} } \mathord{\left/ {\vphantom {{c_{ISF} } {c_{plasma} }}} \right. \kern-\nulldelimiterspace} {c_{plasma} }}$, its value is directly related with the ${{k_{CS \to ISF} } \mathord{\left/ {\vphantom {{k_{CS \to ISF} } {k_{ISF \to CS} }}} \right. \kern-\nulldelimiterspace} {k_{ISF \to CS} }}$ ratio through $f_{plasma}^{u}$ and $f_{ISF}^{u}$ (Eq. 14). This equivalence allows expressing for both $k_{CS \to ISF}$ and $k_{ISF \to CS}$ the same physiological restrictions applied by Cao, Jusko [26] concerning CO. Furthermore, Eq. (13) shows that the ${{k_{CS \to ISF} } \mathord{\left/ {\vphantom {{k_{CS \to ISF} } {k_{ISF \to CS} }}} \right. \kern-\nulldelimiterspace} {k_{ISF \to CS} }}$ ratio is related to the ratio of unbound concentrations at steady-state, which are expected to be equal [35, 43]. The values of $k_{CS \to ISF}$ and $k_{ISF \to CS}$, which are fitted, will account for the actual degree of permeability limitation in the capillary exchange and are expected to compensate for modeling errors, *e.g.* compartment volumes and protein binding.

### Methotrexate cellular exchange

The minimal PBPK model is extended with an ICF compartment as MTX is affected by extensive cellular uptake [1]. Inside cells, methotrexate undergoes an extensive, reversible transformation from its native monoglutamate form into polyglutamate derivatives by folylpolyglutamate synthase (FPGS) [1, 44]. Our model does not provide an explicit description of the polyglutamate forms of methotrexate and neglects their efflux. Since Galivan [45] reports an intracellular polyglutamation exceeding 90%, this value is tentatively taken as the reference value for the ICF bound fraction, described with $f_{ICF}^{b}$ and $f_{ICF}^{u}$ of about 90% and 10%, respectively.

We assume that the total MTX uptake $\dot{m}_{ISF \to ICF}$ can be sufficiently described with a single Michaelis–Menten equation, representing the RFC (*i.e.* Reduced Folate Carrier [1]) transport system, as a function of the unbound ISF concentration:

15 $$\dot{m}_{ISF \to ICF} = \dot{m}_{ISF \to ICF}^{max} \cdot \frac{{c_{ISF}^{u} }}{{K_{50}^{RFC,influx} + c_{ISF}^{u} }}.$$

Similarly, Eq. (16) describes the total MTX efflux $\dot{m}_{ICF \to ISF}$ as a function of unbound ICF concentration:

16 $$\dot{m}_{ICF \to ISF} = \dot{m}_{ICF \to ISF}^{max} \cdot \frac{{c_{ICF}^{u} }}{{K_{50}^{RFC,efflux} + c_{ICF}^{u} }}.$$

The Michaelis influx constant is approximately equal to 5 µM [46], multiplied by $f_{ISF}^{u}$, and since RFC efflux shares significant similarities with RFC uptake [46], $K_{50}^{RFC,efflux}$ is assumed to be equal to $K_{50}^{RFC,influx}$.

Finally, $\dot{m}_{ISF \to ICF}^{max}$ and $\dot{m}_{ICF \to ISF}^{max}$ are the maximum rates exchanged between ISF and ICF and are assumed proportional to the total ICF volume (Eq. 17).

17 $$\begin{gathered} \dot{m}_{ISF \to ICF}^{max} = k_{ISF \to ICF}^{max} V_{ICF} , \hfill \\ \dot{m}_{ICF \to ISF}^{max} = k_{ICF \to ISF}^{max} V_{ICF} , \hfill \\ \end{gathered}$$

where the intensive parameters $k_{ISF \to ICF}^{max}$ and $k_{ICF \to ISF}^{max}$ (min^−1^) are fitted.

Table 1 summarizes all the model parameters, detailing whether (i) they have a fixed assigned value, (ii) are estimated according to the individual features of the patient (“Individualized”), or (iii) are determined via a regression procedure (“Fitted”).

**Table 1:** List of model parameters

Parameter	Description	Value	Units
$V_{plasma}$	Total plasma volume	Individualized	mL
$V_{CS}$	CS plasma volume	Individualized	mL
$V_{RCS}$	RCS plasma volume	Individualized	mL
$V_{HCS}$	HCS plasma volume	Individualized	mL
$V_{ISF}$	ISF volume	Individualized	mL
$V_{ICF}$	ICF plasma volume	Individualized	mL
$Q_{CO}^{plasma}$	Plasma cardiac output	Individualized	mL/min
$Q_{CS \leftrightarrow RCS}^{plasma}$	Renal plasma flow (through renal arteries)	Individualized	mL/min
$Q_{CS \leftrightarrow HCS}^{plasma}$	Hepatic plasma flow (through the hepatic vein)	Individualized	mL/min
$f_{plasma}^{b}$/$f_{plasma}^{u}$	Bound/unbound MTX fraction in plasma	0.42/0.58	–
$f_{ISF}^{b}$/$f_{ISF}^{u}$	Bound/unbound MTX fraction in ISF	0.28/0.72	–
$f_{ICF}^{b}$/$f_{ICF}^{u}$	Bound/unbound MTX fraction in ICF	0.9/0.1	–
$Q_{GFR}$	Glomerular filtration rate	Individualized	mL/min
$K_{50}^{secretion}$	Secretion Michaelis constant	1.3179E-04	mg/mL
$\dot{m}_{secretion}^{max}$	Maximum rate of secretion	Individualized	µg/min
$K_{50}^{reabsorption}$	Reabsorption Michaelis constant	2.6358E-05	mg/mL
$\dot{m}_{reabsorption}^{max}$	Maximum rate of reabsorption	Individualized	µg/min
$Q_{clerance}^{hepatic}$	Overall hepatic clearance	Individualized	mL/min
$K_{CS \to ISF}$	CS → ISF exchange constant	Individualized	mL/min
$k_{CS \to ISF}$	CS → ISF intensive exchange constant	Fitted	min^−1^
$K_{ISF \to CS}$	ISF → CS exchange constant	Individualized	mL/min
$k_{ISF \to CS}$	ISF → CS intensive exchange constant	Fitted	min^−1^
$K_{50}^{RFC,influx}$	Cellular influx Michaelis constant	3.6	µmol/L
$\dot{m}_{ISF \to ICF}^{max}$	Maximum rate of cellular uptake	Individualized	mg/min
$k_{ISF \to ICF}^{max}$	Intensive maximum cellular uptake constant	Fitted	min^−1^
$K_{50}^{RFC,efflux}$	Cellular efflux Michaelis constant	3.6	µmol/L
$\dot{m}_{ICF \to ISF}^{max}$	Maximum rate of cellular efflux	Individualized	mg/min
$k_{ICF \to ISF}^{max}$	Intensive maximum cellular efflux constant	Fitted	min^−1^

## Case study

We employed the experimental dataset of Mei et al. [41] to perform the model identification and validation. The dataset collects the individual information of 98 Chinese patients with PCNSL who underwent intravenous HDMTX treatment in Beijing Tiantan Hospital. A preliminary screening selected only the adults aged ≥ 18 y of the study and allowed removing three outlier patients, *i.e.* patients labeled 580.1, 613.3, and 614.1, who showed plasma MTX concentrations higher than 0.2 µM after more than 150 h following the administration of a 1.5–1.6 g dose. This led to the selection of 84 patients.

For each patient, Mei et al. [41] collected the body weight (44–100 kg), height (146–181 cm), gender (43 men, 41 women), age (27–83 y), data on comedications, as well as the results of laboratory tests such as hematocrit, serum creatinine, blood albumin, and total blood proteins. Most of the reported data were measured periodically. The population presents a wide range of degrees of renal function, as their estimated GFR values range from 35 mL/min to 162 mL/min (39–150 mL/min/1.73m^2^), including patients with mild/moderate renal impairment.

Patients received from 1 to 17 administrations of the drug, over a time period from 36 h to 352 d, for a grand total of 396 infusions. The dataset reports the amount of each administration (1–9 g, or 0.62–5.16 g/m^2^), the time of administration, and the infusion duration (0.32–7.71 h). We assume that the administered infusion rate was constant over the reported duration. Following administration, Mei et al. [41] measured plasma MTX concentrations every day, until the concentration fell below 0.05 µM, yielding 657 measurements ($N_{tot}^{exp}$).

As discussed in the Methods section, the number of adaptive population parameters was kept as low as possible ($k_{CS \to ISF}$, $k_{ISF \to CS}$, $k_{ISF \to ICF}^{max}$, $k_{ICF \to ISF}^{max}$). Their value is computed numerically (*i.e.* identified) through a non-linear regression procedure (Eq. 18, see [27]) against a subset of the experimental data [41] comprising 56 adult patients from Mei et al. [41]. These patients and the remaining twenty-eight patients feature similar distributions of body weight, gender, GFR, total blood proteins, and hematocrit.

18 $$\mathop {\min }\limits_{{k_{CS \to ISF} ,k_{ISF \to CS} ,k_{ISF \to ICF}^{max} ,k_{ICF \to ISF}^{max} }} \left\{ {ObjFun} \right\}.$$

We estimated $ObjFun$, the objective function, as the mean squared logarithmic errors between each experimental point $c_{CS,ij}^{exp}$ and their corresponding simulated value $c_{CS,ij}^{sim}$:

19 $$ObjFun = \frac{1}{{N_{tot}^{exp} }}\sum\limits_{i = 1}^{{N_{P} }} {\sum\limits_{j = 1}^{{N_{i}^{exp} }} {\left[ {\log \left( {c_{CS,ij}^{exp} } \right) - \log \left( {c_{CS,ij}^{sim} } \right)} \right]^{2} } } .$$

## Results

The regression procedure entailed a number of preliminary consecutive 4-dimension grid searches in a wide range of values (*i.e.* 1.E-08-1.E0), to identify a suitable starting point for an unconstrained minimization of the objective function. We carried out a 500-sample bootstrap analysis by resampling the 56 patients of the identification dataset and running a non-linear regression for each bootstrap sample. We obtained an approximately normal distribution for each fitted parameter and identified their mean value and standard deviation (Table 2). The mean identified parameters lead to an objective function of 0.8568 for the identification dataset.

**Table 2:** Regressed parameters values

Parameter	Investigated range	Initial value	Mean fitted value	Standard deviation	Units
$k_{CS \to ISF}$	1.E-08–1.E0	2.1544E-02	1.5782E-03	2.3759E-04	min^−1^
$k_{ISF \to CS}$	1.E-08–1.E0	2.1544E-02	5.1829E-03	2.6271E-04	min^−1^
$k_{ISF \to ICF}^{max}$	1.E-08–1.E0	3.1623E-07	2.4088E-07	2.9131E-08	min^−1^
$k_{ICF \to ISF}^{max}$	1.E-08–1.E0	3.1622E-06	5.6171E-06	1.1798E-06	min^−1^
$ObjFun$	–	1.2839E0	8.5684E-01	1.0909E-01	–

The identified parameters allow to numerically assess the ${{k_{CS \to ISF} } \mathord{\left/ {\vphantom {{k_{CS \to ISF} } {k_{ISF \to CS} }}} \right. \kern-\nulldelimiterspace} {k_{ISF \to CS} }}$ ratio, which is equal to 0.3045 and might result from the compensation of errors of CS/ISF protein binding and volume estimates. The ratio agrees with the expected order of magnitude, *i.e.* around unity.

**Fig. 2:**
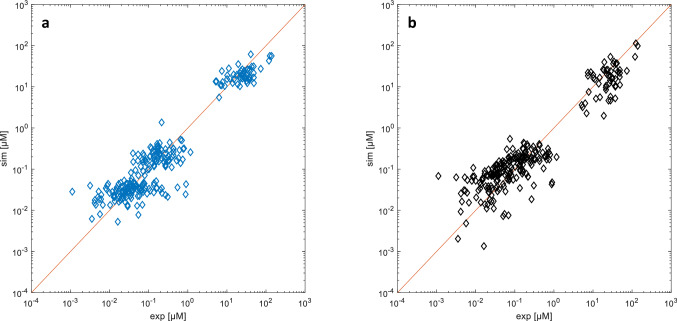
Model predicted vs observed concentration plots for **a** our minimal PBPK model (ObjFun = 0.8175) and **b** the 2-compartment pharmacokinetic model of Mei et al. [41] (ObjFun = 1.0799). For each experimental plasma concentration (*x*-axis), the diagrams show the corresponding simulated value (y-axis)

The simulation of the validation dataset featuring the remaining 28 patients yielded an objective function of 0.8175 (Fig. 2a), which is close to the one produced by the identification procedure (0.8568).

These results are compared with the simulations of a 2-compartment pharmacokinetic model used in many literature MTX studies, applied by Mei et al. [41] to the same experimental dataset (Fig. 2b). For the same dataset used to validate our minimal PBPK model, the 2-compartment simulation yields an objective function of 1.0799.

**Fig. 3:**
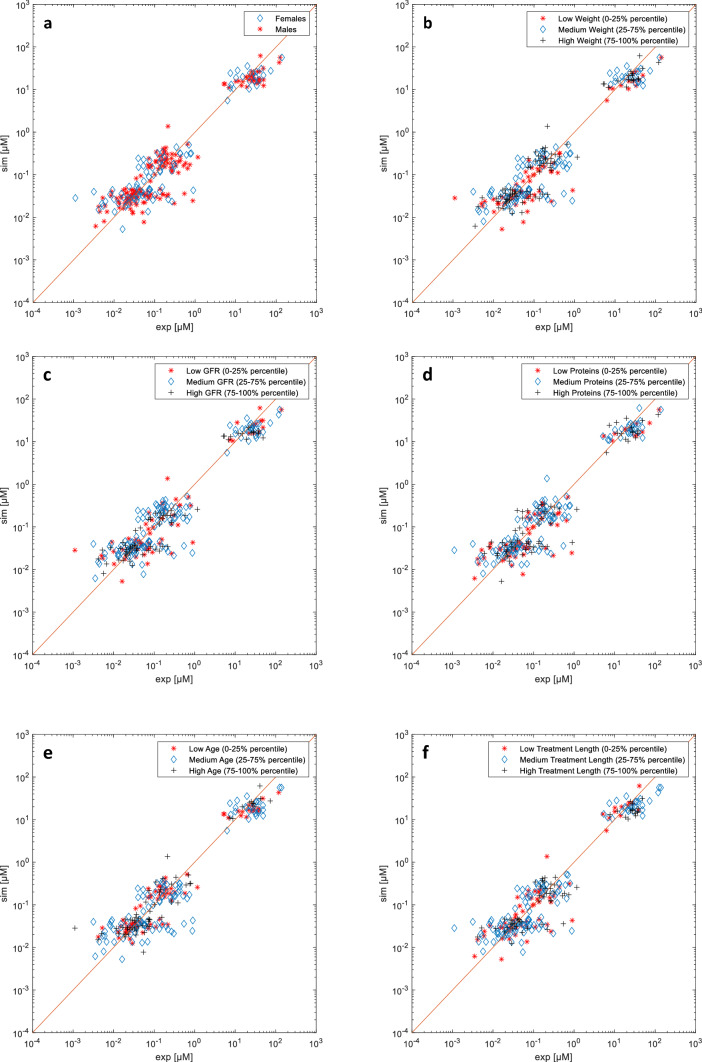
Model predicted vs observed concentration plots that highlight the conditions of each experimental point, according to **a** gender, **b** body weight, **c** GFR, **d** total blood proteins, **e** age, **f** length of treatment

Figure 3a-f shows an analysis performed according to the specific conditions of the patients at each specific time, for sex, body weight, GFR, blood protein levels, age, and length of treatment. All the characteristic values are classified according to their distribution as low (0–25 percentiles), medium (26–75 percentiles), and high (76–100 percentiles).

The predicted overall MTX clearance, estimated as $\left( {\dot{m}_{excretion}^{renal} + \dot{m}_{elimination}^{hepatic} } \right)/c_{CS}^{u}$, approximately follows a normal distribution (mean 92.5 ml/min, range 44–149 ml/min, coefficient of variation 21%) for the 84 patients in the dataset.

**Fig. 4:**
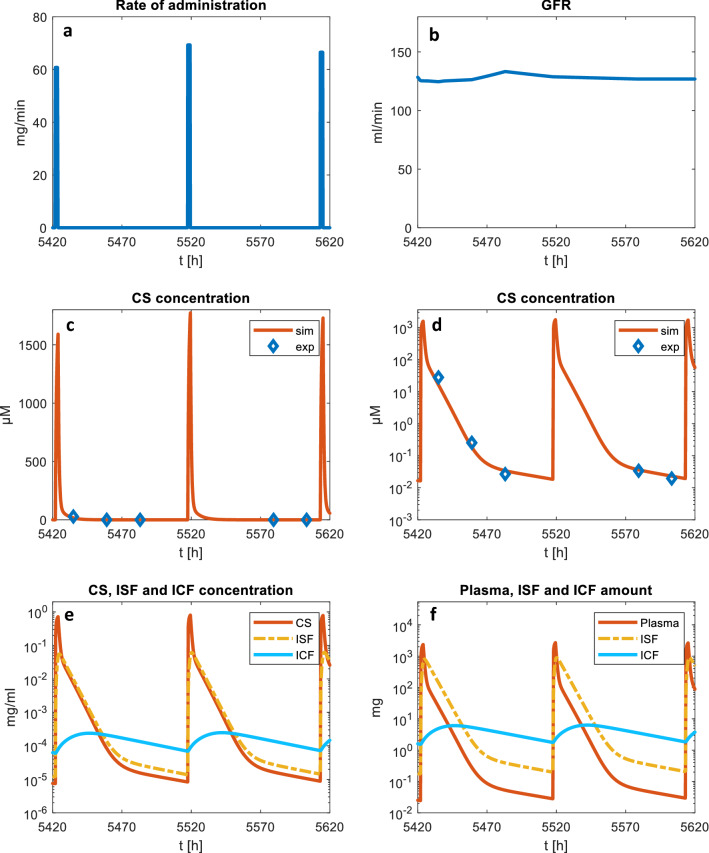
Simulated curves for patient 630.3 with median error (ObjFun = 0.5416): **a** infusion rates, **b** estimated GFR, **c**-**d** simulated and experimental CS concentrations (in linear and logarithmic coordinates), **e** comparison of CS, ISF and ICF concentrations, **f** comparison of drug amounts in the overall plasma (CS + RCS + HCS), ISF, and ICF

Figure 4 shows the simulated curves for a specific section of the clinical data of patient 630.3, whose objective function (0.5416) corresponds to the median value within the validation dataset. The 200-h section includes three close, consecutive infusions and is deemed as representative of the whole dataset and useful to discuss the consistency of model predictions and their limitations.

Despite the wide range of experimental concentrations, as can be seen from both Fig. 4c and d, the drug levels measured by Mei et al. [41] never capture the peak following HDMTX administration. In fact, while the drug administrations started between 9:45 AM and 11:15 PM, and finished in most cases before midnight (at most at 3:30 AM of the following day), the blood samples for MTX analysis were always taken between 5 AM and 6 AM, *i.e.* hours later.

The model describes ICF concentrations that reach peaks of 0.2–0.3 µg/mL, *i.e.* 0.44–0.66 µM. Indeed, the ICF compartment acts as an MTX reservoir that becomes significant when plasma MTX levels fall below 0.1 µM.

At concentrations lower than 0.1 µM, the simulations show a steady and very slow decrease of ICF concentration. As a result, the linear trend (in logarithmic coordinates of Fig. 4d) of plasma concentration is interrupted at about 0.1 µM and a new quasi-linear (*i.e.* approximately exponential in linear coordinates) decrease can be observed, which is much slower, with a half time of about 40 h.

## Discussion

Four population parameters of the model are identified with a bootstrap analysis and non-linear regressions of individual experimental data that describe a population of patients suffering from PCNSL. The values of the $k_{CS \to ISF}$ and $k_{ISF \to CS}$ constants determine low $K_{CS \to ISF} /Q_{CO}^{plasma}$ fractions, in the 0.4–0.8% range, which suggests permeability-limited capillary exchange and distribution [26]. Despite its molecule being relatively small (454 g/mol), MTX is hydrophilic and has limited lipid solubility [15]. As for $k_{ICF \to ISF}^{max}$ and $k_{ISF \to ICF}^{max}$, the maximum efflux rate $k_{ICF \to ISF}^{max}$ is higher than the cellular uptake rate $k_{ISF \to ICF}^{max}$, in agreement with [46].

The model is validated against a different set of patients from the same dataset. Figure 2 shows a wide distribution across the bisector. Indeed, the literature reports a very large intra- and inter-individual variability in the case of HDMTX treatment [7–9], and also Mei et al. [41] label this variability as “extremely large”. Despite this, results suggest that the identified parameters are capable of capturing the average pharmacokinetic behavior of the diseased population. The comparison with a pharmacokinetic model used in several literature studies (Fig. 2b) also shows that, despite the rigid prior estimation of compartment volumes and clearance flows, the minimal PBPK model predictions appear to better account for the wide experimental variability of the dataset.

We investigated the model predictions against sex, weight, GFR, protein levels, or age (Fig. 3a-e) and found no indication of any clear systematic error. Remarkably, the model does not present any significant bias in a wide range of degrees of renal function, with estimated GFR values ranging from 35 mL/min to 162 mL/min (39  mL/min/1.73 m^2^ to 150 mL/min/1.73 m^2^) (Fig. 3c). This is expected to favor the application of the model to special populations with different characteristics, such as people with renal impairment. Conversely, when treatment length is medium (40–111 days) or high (111–325 days), model predictions appear to slightly underestimate the higher concentration values (Fig. 3f). This might indicate renal impairment arising with repeated administrations of HDMTX, as reported by [47]. Despite these findings, however, we did not impose a kidney function decline in our model. While the hypothesis of renal impairment is consistent with reports from the literature [47], the observed effect might be due to other factors, *e.g.* comedications such as dexamethasone, or also to a selection bias (patients who undergo longer treatments are ill patients who have not recovered yet despite multiple HDMTX cycles).

Finally, we described an intracellular fluid compartment characterized by slow saturable exchanges, which determines the prediction of a slow MTX decrease at low plasma concentrations (in the 0.001–0.1 µM range). The overall predicted behavior appears generally consistent with the reference trend of MTX decay in Abelson et al. [48]. Further studies are recommended to improve the modeling of cellular uptake, efflux, and other intracellular phenomena.

We presented the development, identification, and validation of a minimal PBPK model of i.v. HDMTX methotrexate for adult patients. The model lumps the organs and tissues of the human body, and explicitly describes distinct compartments for plasma, interstitial fluid, and intracellular fluid. Since renal excretion is the main elimination route for methotrexate, the model is extended with a detailed description of renal excretion according to an anatomical and physiological approach. The model accounts for the specific characteristics and clinical data of each patient. Individual features such as body weight, height, gender, age, hematocrit, and serum creatinine are used to estimate a priori the pharmacokinetic parameters of each patient. This allows obtaining individualized predictions of MTX pharmacokinetics as a function of i.v. HDMTX administrations.

The model was developed using a dataset of Chinese patients with PCNSL and can be generalized to other populations by employing a suitable equation to estimate the GFR and by describing the different features of the target population, *e.g.* body volumes and protein binding.

Future developments should attempt to account for the residual intra- and inter-individual variability, especially by improving the estimation of volumes and exchanges as a function of further patient’s features. In addition, model identification and validation should be replicated using different experimental datasets, possibly including patients with severe renal impairment, describing peak concentrations, and focusing on different populations and therapeutic protocols.

We believe that our model can be used to propose better individualized dosages and schedules of HDMTX, support the therapeutic monitoring of patients, and lead to better clinical treatment.
